# A Randomized Controlled Trial Comparing Laser Versus Open Surgical Approaches in the Management of Fistula-in-Ano at a Tertiary Care Center

**DOI:** 10.7759/cureus.90471

**Published:** 2025-08-19

**Authors:** Kamal Raj Patel, Ramendra K Jauhari, Priyesh Shukla

**Affiliations:** 1 General Surgery, Ganesh Shankar Vidyarthi Memorial (GSVM) Medical College, Kanpur, IND; 2 General Surgery, Government Medical College and Super Facility Hospital, Azamgarh, IND

**Keywords:** colorectal surgery, fistula-in-ano, laser surgery, open surgery, patient satisfaction, postoperative pain, randomized controlled trial, recurrence, sphincter preservation, wound healing

## Abstract

Background: Fistula-in-ano remains a challenging surgical entity due to its high recurrence rate and potential risk of incontinence. This study evaluates and compares the efficacy, safety, and patient-centered outcomes of laser surgery and open surgical procedures.

Methods: This prospective randomized controlled trial (RCT) (ClinicalTrials.gov identifier: NCT07083778) included 100 patients diagnosed with intersphincteric or transsphincteric fistula-in-ano at Ganesh Shankar Vidyarthi Memorial (GSVM) Medical College, Kanpur, India. Patients were randomized using the even-odd method into two groups: open surgery (n=50) and laser surgery (n=50). Assessed parameters included operative time, postoperative pain (visual analog scale), hospital stay, return to daily activity, healing time, recurrence, incontinence (Wexner score), complications, and patient satisfaction.

Results: The laser group showed significant advantages in terms of postoperative pain, with 35 patients (70%) reporting only mild pain compared to 15 patients (30%) in the open surgery group (p<0.01). Hospital stay was shorter in the laser group (1.5 ± 0.5 days) compared to the open group (3.8 ± 1.2 days; p<0.001), and return to normal activity was quicker (4.96 ± 0.91 vs. 9.96 ± 0.74 days; p<0.001). However, open surgery demonstrated superior clinical outcomes. Complete healing at three months was achieved in 41 patients (82%) in the open group vs. 35 patients (70%) in the laser group (p=0.16). Recurrence was observed in 15 patients (30%) in the laser group and nine patients (18%) in the open group, approaching statistical significance (p=0.06). Wound infection occurred more frequently in the open group with eight cases (15%) compared to three cases (5%) in the laser group (p=0.034), though all were minor. Incontinence was reported in two patients (5%) and anal stenosis in one patient (2%), all within the open group. Reoperation was more commonly required in the laser group (15 patients, 30%) compared to the open group (nine patients, 18%). Overall, patient satisfaction was slightly higher in the open surgery group (mean score: 6.98 ± 1.96) than in the laser group (6.34 ± 2.29).

Conclusion: While laser surgery offers faster recovery and reduced postoperative morbidity, open surgery remains the more definitive treatment option due to its superior long-term outcomes. Surgical decision-making should be individualized based on fistula complexity and patient-specific factors.

## Introduction

Fistula-in-ano is a chronic anorectal condition characterized by an abnormal communication between the anal canal and the perianal skin. It typically develops following an anal gland infection that progresses to abscess formation, which, upon rupture or inadequate drainage, evolves into a fistula tract [1]. The underlying pathophysiology involves glandular obstruction, infection, abscess formation, and the development of persistent epithelialized tracts, often resulting in recurrent infections, pain, and purulent discharge [2].

Fistulas are broadly classified into simple and complex types based on their anatomical relationship to the anal sphincters. Simple fistulas typically involve a single tract with minimal sphincter involvement and are generally amenable to standard surgical treatment. In contrast, complex fistulas may involve multiple tracts, horseshoe extensions, or high transsphincteric pathways, with increased risks of recurrence and incontinence following treatment [3]. Clinically, patients may present with persistent perianal pain, swelling, discharge of pus or blood, pruritus, and discomfort during sitting or walking. These symptoms can significantly impair personal hygiene, social interactions, and overall quality of life [4].

Surgical intervention remains the cornerstone of management. Traditional methods, such as fistulotomy, fistulectomy, the ligation of the intersphincteric fistula tract (LIFT) procedure, and seton placement, have demonstrated efficacy, particularly in simple fistulas [5,6]. However, these procedures may be associated with prolonged recovery, potential sphincter injury, and fecal incontinence. Managing complex fistulas presents additional challenges in achieving durable healing while preserving continence.

In recent years, minimally invasive options, such as laser-assisted fistula treatment (LAFT), have gained attention. This technique employs radial-emitting laser fibers to ablate the fistulous tract internally, promoting closure with minimal trauma to surrounding tissues [7,8]. The reported advantages of LAFT include reduced postoperative pain, faster recovery, improved sphincter preservation, and higher patient satisfaction.

Given the chronic nature of the condition and its significant impact on quality of life, effective surgical treatment is essential - not only to resolve symptoms but also to prevent complications, such as recurrent abscesses, secondary tract formation, and incontinence [9,10]. Selecting a treatment strategy that minimizes sphincter injury is critical in preserving long-term continence, particularly in complex fistulas [11].

This randomized controlled trial (RCT) was conducted at a tertiary care center to compare the efficacy and safety of laser versus open surgical approaches in patients with intersphincteric and transsphincteric fistula-in-ano. The primary objectives were to evaluate healing time, recurrence, and postoperative complications. Secondary objectives included assessing patient satisfaction and identifying predictors of poor outcomes. This study aims to generate robust clinical evidence to guide optimal surgical decision-making, balancing efficacy, recurrence risk, and continence preservation [12,13].

## Materials and methods

Study design and setting

This prospective RCT was conducted at the Department of Surgery, GSVM Medical College, Kanpur, India, from April 2024 to March 2025. Ethical approval was obtained from the Institutional Ethics Committee for Biomedical and Health Research (Protocol No. EC/BMHR/2024/87). This study was registered on ClinicalTrial.gov with the identifier NCT07083778. Patients were randomized into two groups using an even-odd allocation method.

Sample size calculation

The sample size was determined using a two-proportion comparison method. Based on previous literature, we assumed a recurrence rate of 30% for laser surgery and 15% for open surgery. To detect this difference with 80% power and a two-sided significance level of 5% (α = 0.05), the minimum required sample size was calculated to be 45 patients per group.

Inclusion and exclusion criteria

To accommodate possible attrition, we included 50 patients in each group, resulting in a total sample size of 100 participants. The inclusion and exclusion criteria are summarized in Table 1. Patients presenting to the outpatient department (OPD) who met the inclusion criteria were screened and enrolled.

**Table 1:** Inclusion and exclusion criteria HIV: Human immunodeficiency virus

Inclusion Criteria	Exclusion Criteria
Age > 18 years	Inflammatory bowel disease (IBD)
Diagnosed with intersphincteric or transsphincteric fistula-in-ano	Tuberculosis
Provided written informed consent for surgery	Anorectal malignancy
	HIV-positive status
	Pregnancy

Surgical procedures

This was a prospective, parallel-arm RCT conducted at a tertiary care center. Patients diagnosed with fistula-in-ano who met the inclusion and exclusion criteria were enrolled after obtaining informed consent. Randomization into two treatment arms was performed using the odd-even method based on the order of registration: patients with odd registration numbers were assigned to the open surgery group (Group A), while those with even numbers were allocated to the laser surgery group (Group B).

**Figure 1:**
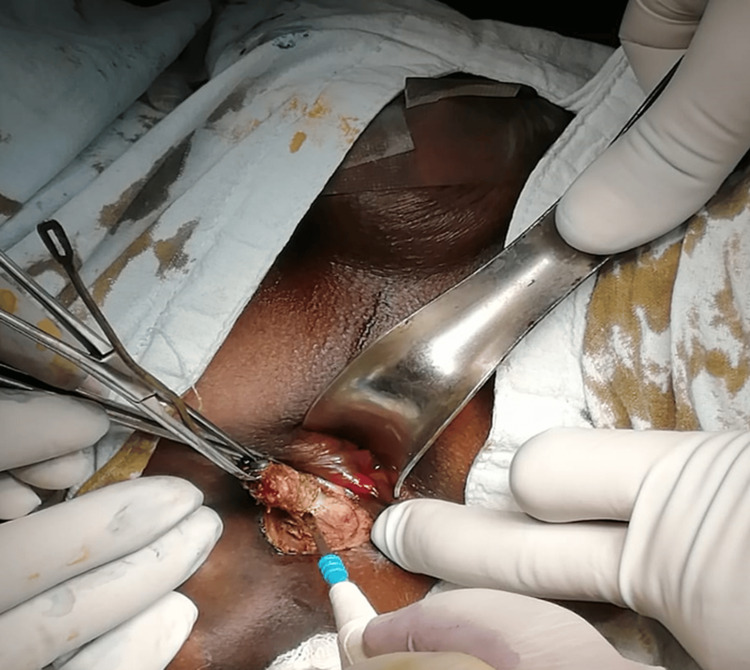
Intraoperative photograph showing fistulectomy being performed The external fistulous tract is excised en bloc with surrounding granulation tissue. Adequate exposure is maintained using a posterior anal retractor, and electrocautery is used for hemostasis.

**Figure 2:**
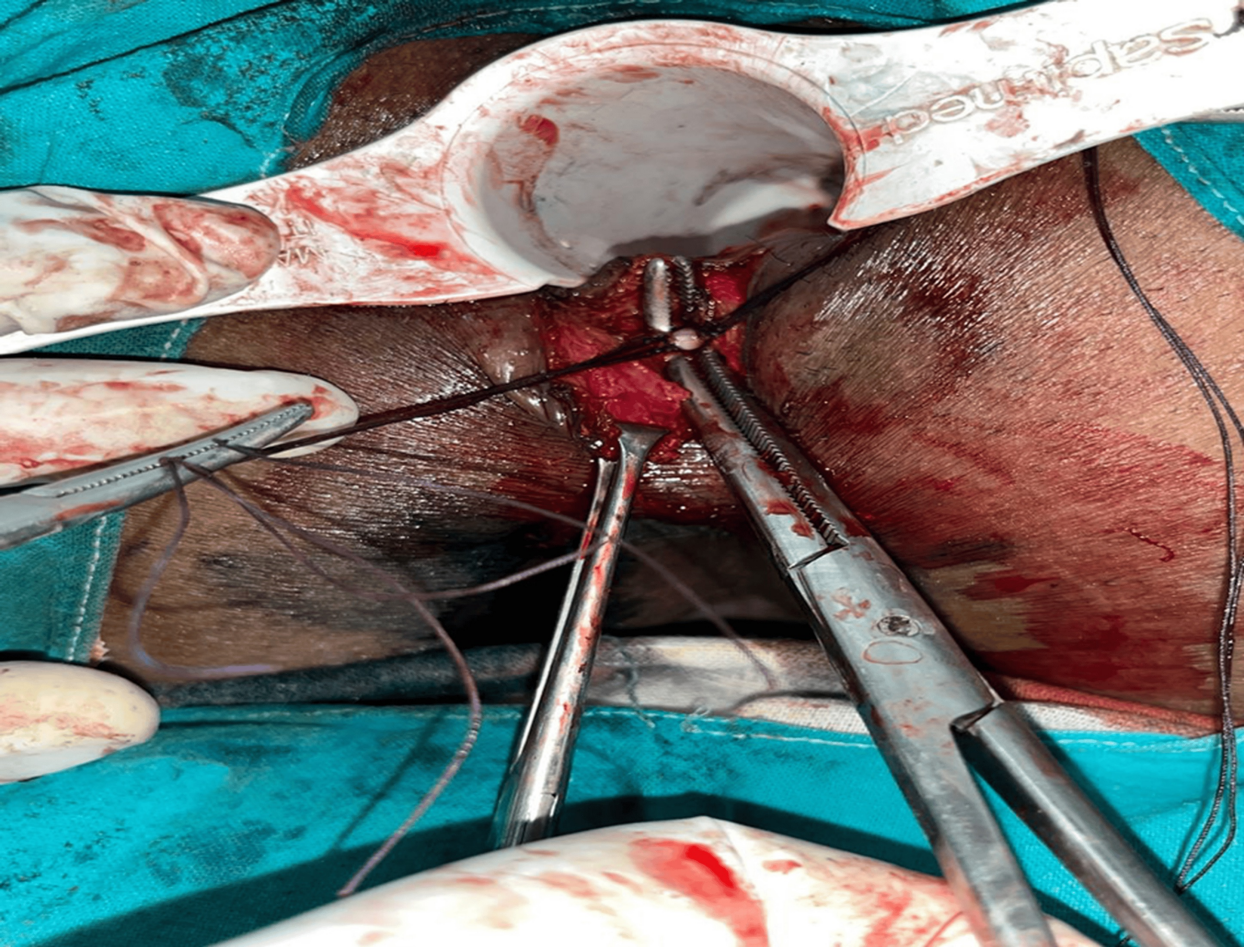
Intraoperative image showing the ligation of the intersphincteric fistula tract (LIFT) procedure The fistulous tract is being ligated near the internal opening using absorbable sutures, with careful preservation of the surrounding sphincter complex.

Open surgery group (n = 50): Patients underwent one of the following procedures: fistulectomy in 30 patients (60%) (Figure 1), fistulotomy in 10 patients (20%), or ligation of the intersphincteric fistula tract (LIFT) in 10 patients (20%) (Figure 2), depending on fistula anatomy and surgeon preference.

**Figure 3:**
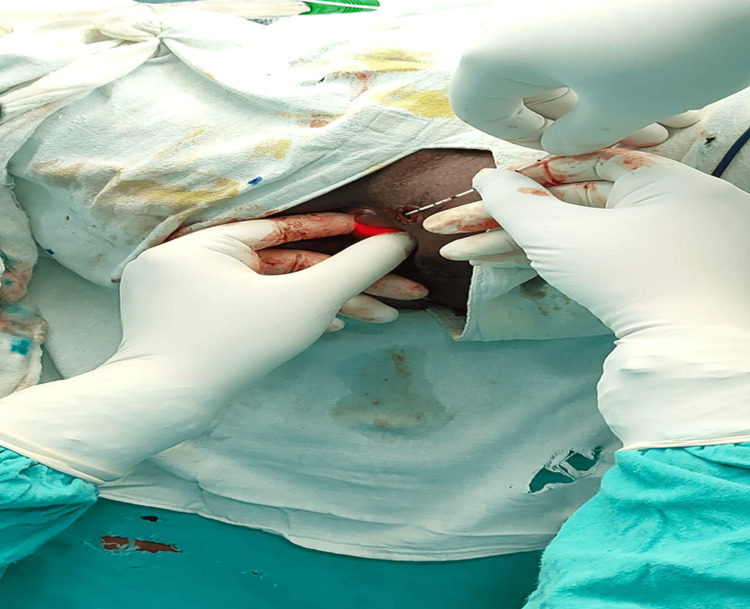
Intraoperative image showing the insertion of a radial laser fiber through the external opening during laser-assisted fistula treatment The illuminated tract confirms accurate placement of the laser probe along the fistulous path.

All patients in the laser group (n = 50) underwent endoscopic laser ablation using a 1,470 nm radial-emitting diode laser system (Figure 3). This system delivers controlled radial energy within the fistulous tract, leading to its coagulation and collapse while minimizing injury to surrounding tissues. The procedure was performed under spinal anesthesia, and care was taken to ensure uniform energy delivery along the entire tract, optimizing healing and reducing postoperative complications.

Statistical analysis

Data analysis was performed using SPSS (version 13.0; IBM Corp., Armonk, NY). Categorical variables were compared using the chi-square test. Continuous variables were presented as mean ± standard deviation. A p-value <0.05 was considered statistically significant.

## Results

Demographic and clinical characteristics

This prospective randomized controlled trial enrolled 100 patients with fistula-in-ano, equally allocated into two groups: laser surgery (n = 50, 50%) and open surgery (n = 50, 50%), using an even-odd randomization method at enrollment. The mean age of participants was 42.5 ± 10.2 years. Most patients were aged 35-50 years (n = 55, 55%), followed by 51-65 years (n = 25, 25%) and 18-34 years (n = 20, 20%). A male predominance was observed with 78 males (n = 78, 78%) and 22 females (n = 22, 22%), consistent with global epidemiological trends. Occupationally, 40 patients (n = 40, 40%) were manual laborers, 35 (n = 35, 35%) were office workers, and 25 (n = 25, 25%) were unemployed or engaged in domestic duties. Socioeconomic assessment revealed that individuals from lower-income backgrounds often experienced delayed access to care. A majority (n = 60, 60%) resided in rural areas, while 40 (n = 40, 40%) lived in urban settings, suggesting geographical disparities in healthcare access. Table 2 presents the baseline characteristics of participants undergoing laser versus open surgery for fistula-in-ano.

**Table 2:** Baseline characteristics of participants undergoing laser versus open surgery for fistula-in-ano (N = 100) Values are expressed as frequency (percentage) unless otherwise specified. External opening positions refer to the anatomical clock face in the lithotomy position (6 o’clock = posterior midline; 12 o’clock = anterior midline). ENDO-LASER: Endoscopic radial-emitting 1470 nm diode laser procedure; LIFT: Ligation of the intersphincteric fistula tract. “Not Applicable” is used where a procedure was not performed in the respective group.

Characteristic	Categories	Laser Surgery (n = 50)	Open Surgery (n = 50)
Age, yr	18–34	10 (20)	10 (20)
	35–50	28 (56)	27 (54)
	51–65	12 (24)	13 (26)
Gender	Male	39 (78)	39 (78)
	Female	11 (22)	11 (22)
Occupation	Manual	20 (40)	20 (40)
	Office	17 (34)	18 (36)
	Unemployed	13 (26)	12 (24)
Residence	Rural	30 (60)	30 (60)
	Urban	20 (40)	20 (40)
Duration of symptoms	< 6 mo	18 (36)	17 (34)
	6–12 mo	22 (44)	23 (46)
	> 12 mo	10 (20)	10 (20)
Fistula type	Intersphincteric	28 (56)	27 (54)
	Transsphincteric	22 (44)	23 (46)
External opening	6 o’clock	25 (50)	25 (50)*
	12 o’clock	10 (20)	10 (20)*
	3 & 9 o’clock	15 (30)	15 (30)*
Number of tracts	Single	42 (84)	38 (76)
	Multiple	8 (16)	12 (24)
Procedure performed	Laser (ENDO-LASER)	50 (100)	Not Applicable
	Fistulectomy	Not Applicable	30 (60)
	Fistulotomy	Not Applicable	10 (20)
	LIFT	Not Applicable	10 (20)
Mean operative time, min	Minutes	40	60

Symptom duration varied, with 45 patients (n = 45, 45%) symptomatic for 6-12 months, 35 (n = 35, 35%) for less than six months, and 20 (n = 20, 20%) for more than a year, indicating chronic disease in many cases. MRI and clinical evaluation identified 55 intersphincteric (n = 55, 55%) and 45 transsphincteric (n = 45, 45%) fistulas. The most common external opening was at the 6 o’clock position (n = 50, 50%), followed by 3 o’clock and 9 o’clock (n = 30, 30%) and 12 o’clock (n = 20, 20%). Most patients (n = 80, 80%) had single-tract fistulas, while 20 (n = 20, 20%) had multiple tracts, including 15 with secondary extensions (n = 15, 15%) and 10 with associated abscess cavities (n = 10, 10%), adding to the complexity of management.

All patients underwent either endoscopic radial-emitting diode laser ablation or open surgery, which included fistulectomy (n = 30, 30%), fistulotomy (n = 10, 10%), and the LIFT procedure (n = 10, 10%). The mean operative time was significantly lower in the laser group (40 ± 10 minutes) compared to the open surgery group (60 ± 15 minutes), highlighting the procedural efficiency of the laser technique (p < 0.05).

Primary endpoints

Table 3 presents the primary endpoints.

**Table 3:** Primary endpoint comparison VAS: Visual analog scale

Parameter	Laser Surgery (n=50)	Open Surgery (n=50)
Mild Pain (VAS 1-3)	70%	30%
Hospital Stay (days)	1.5 ± 0.5	3.8 ± 1.2
Return to Activity (days)	4.96 ± 0.91	9.96 ± 0.74

Postoperative pain

Postoperative pain significantly affects both the recovery trajectory and overall patient satisfaction. Based on a standardized visual analog scale (VAS), 35 patients (70%) in the laser surgery group reported only mild postoperative pain, compared to 15 patients (30%) in the open surgery group. Conversely, severe pain was experienced by 10 patients (20%) in the open surgery group, while only two patients (5%) in the laser group reported similar discomfort. These findings demonstrate a statistically significant reduction in postoperative pain with laser surgery (p < 0.01).

Hospital stay duration

**Figure 4:**
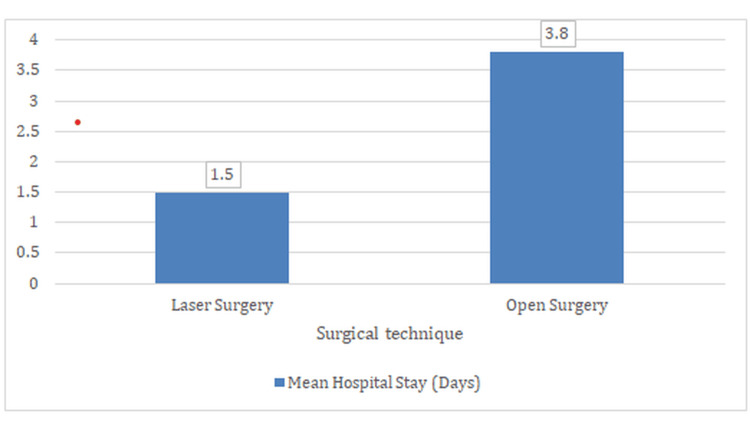
Comparison of mean hospital stay in days between patients undergoing laser surgery and open surgery for fistula-in-ano

One of the notable advantages of laser surgery was a significantly shorter hospital stay (Figure 4). Patients undergoing laser treatment had an average hospital stay of 1.5 ± 0.5 days, in contrast to 3.8 ± 1.2 days in the open surgery group. This difference was highly statistically significant (p < 0.001), highlighting the reduced postoperative burden associated with minimally invasive techniques.

Return to normal activity

**Figure 5:**
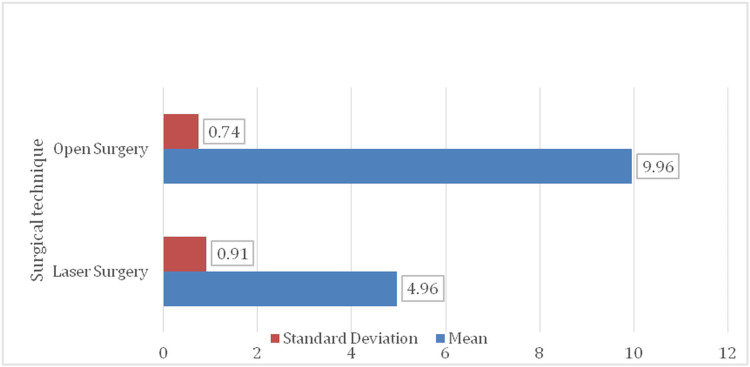
Bar chart for return-to-normal activity (in days) (N = 100)

The duration required to resume normal daily activities was significantly shorter in the laser surgery group (Figure 5). Patients treated with laser resumed routine functions in 4.96 ± 0.91 days, compared to 9.96 ± 0.74 days in the open surgery group. This difference was statistically significant, as confirmed by an independent samples t-test. Levene’s test for equality of variances showed no significant difference (F = 0.053, p = 0.819), validating the use of the t-test. These findings underscore the clinical and statistical advantage of laser surgery in facilitating quicker postoperative recovery.

Secondary endpoints

Table 4 presents the secondary endpoints.

**Table 4:** Secondary endpoint comparision

Parameter	Laser Surgery (n=50)	Open Surgery (n=50)
Healing <3 Months	35 (70%)	41 (82%)
Recurrence	15 (30%)	9 (18%)
Incontinence	0%	2 (5%)
Anal Stenosis	0%	1 (3%)
Wound Infection	3 (5%)	8 (15%)
Reoperation Required	15 (30%)	9 (18%)
Satisfaction Score (/10)	6.34 ± 2.29	6.98 ± 1.96

Healing outcomes

**Figure 6:**
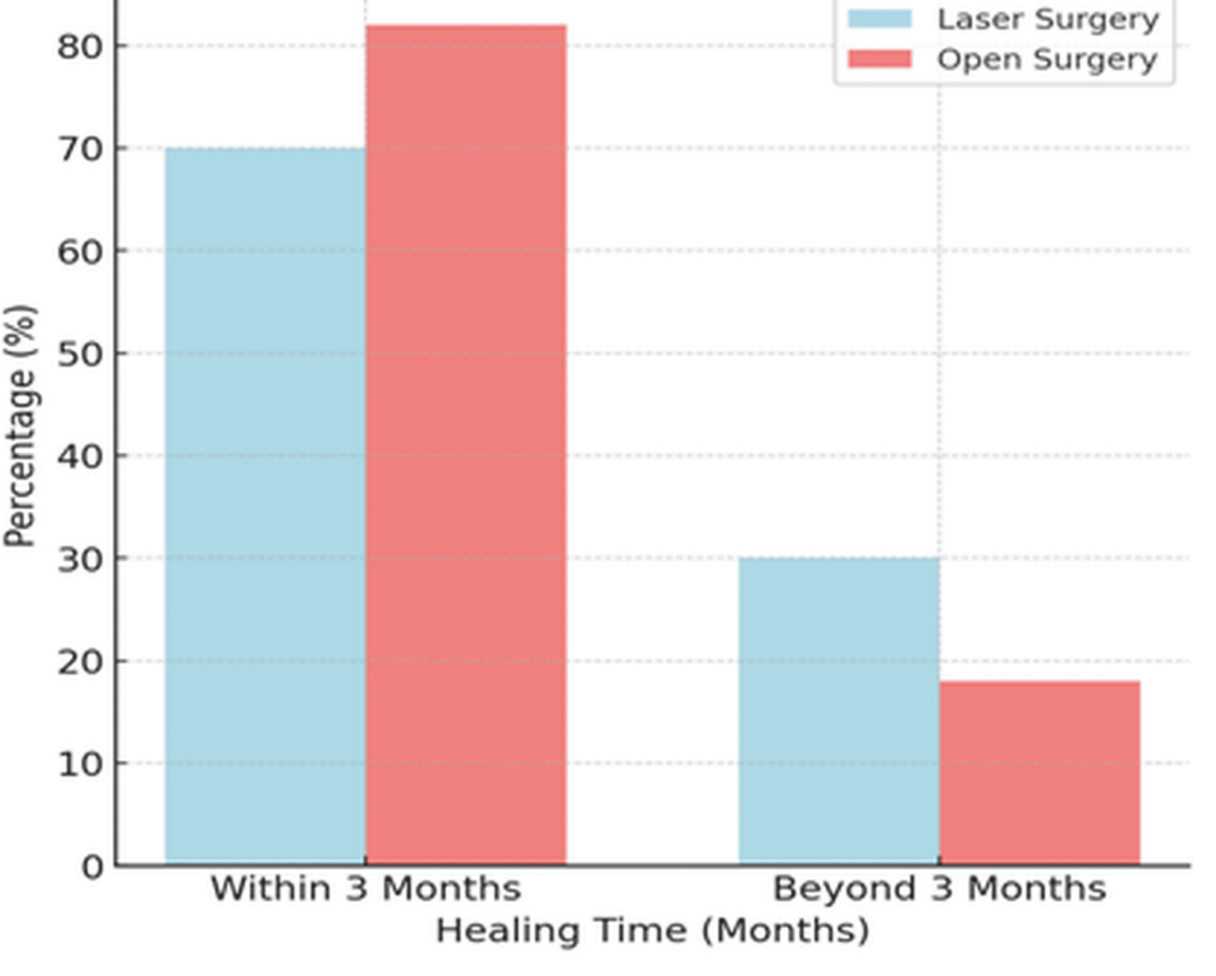
Bar chart for complete healing time (N = 100)

At three months postoperatively, complete healing was observed in 41 patients (82%) in the open surgery group and 35 patients (70%) in the laser surgery group (Figure 6). Although this difference did not reach statistical significance (p = 0.16), it suggests a clinically meaningful trend favoring open surgery - likely attributable to more thorough tract excision and lower rates of residual disease.

Recurrence rate

**Figure 7:**
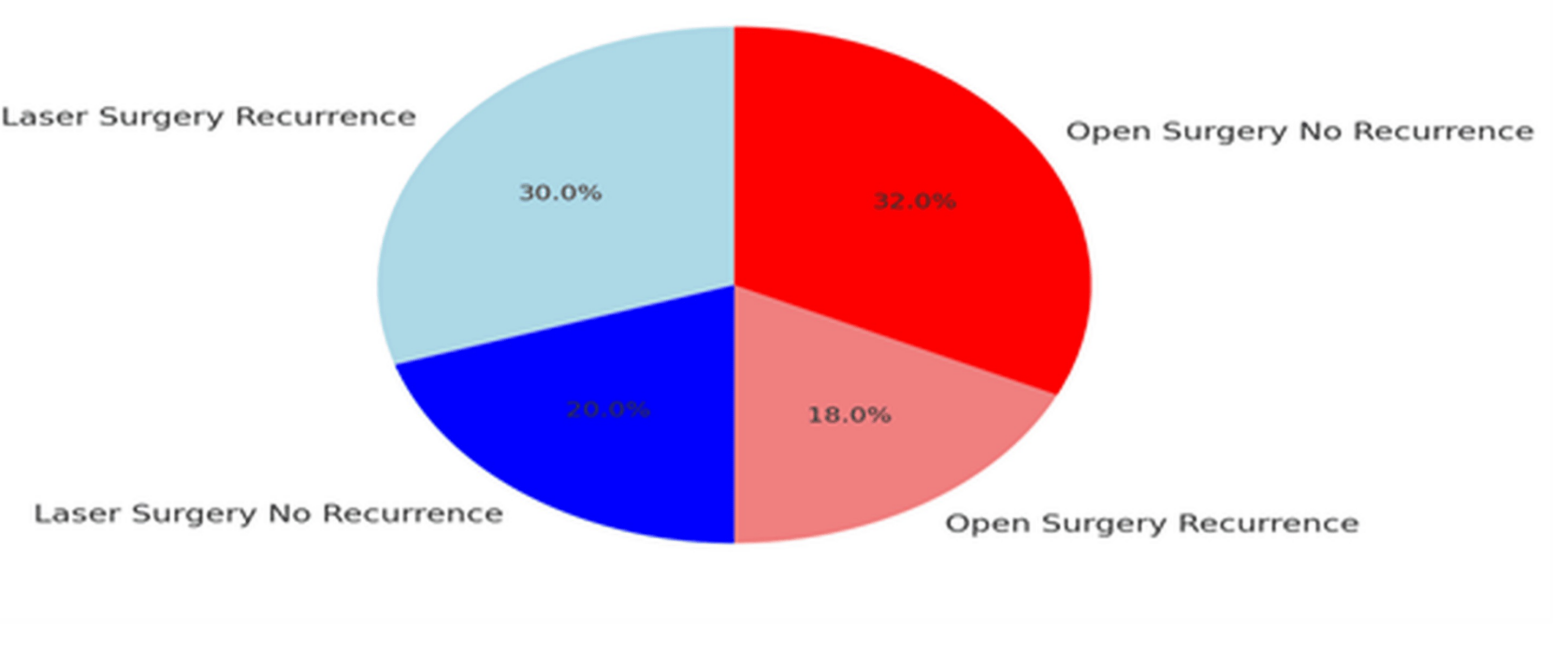
Pie chart for the recurrence rate (>6 months post surgery) (N = 100) Red segment: Open surgery – no recurrence (32%, 32 patients); Pink segment: Open surgery – recurrence (18%, 18 patients); Blue segment: Laser surgery – no recurrence (20%, 20 patients); Light blue segment: Laser surgery – recurrence (30%, 30 patients)

Recurrence occurred in 15 patients (30%) in the laser surgery group, and nine patients (18%) in the open surgery group (Figure 7). While the difference approached statistical significance (p = 0.06), the trend suggests a higher likelihood of recurrence following laser treatment, possibly due to limited excisional clearance of complex tracts.

Anal incontinence

**Figure 8:**
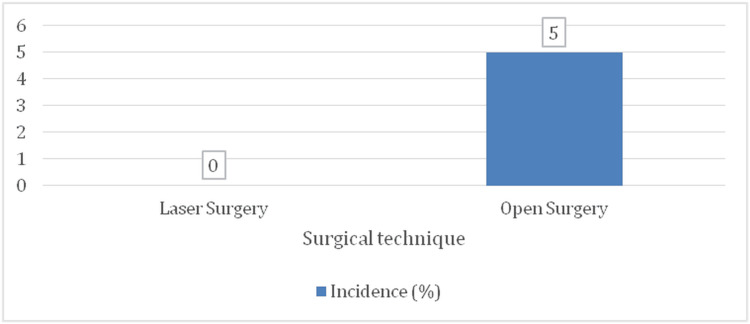
Bar chart for the incidence of anal incontinence (N = 100)

No cases of anal incontinence were reported in the laser surgery group (0 patients), whereas two patients (5%) in the open surgery group experienced mild fecal incontinence (Figure 8). Although this difference was not statistically significant (p = 0.070), it remains notable due to the potential impact on patients' quality of life.

Anal stenosis

**Figure 9:**
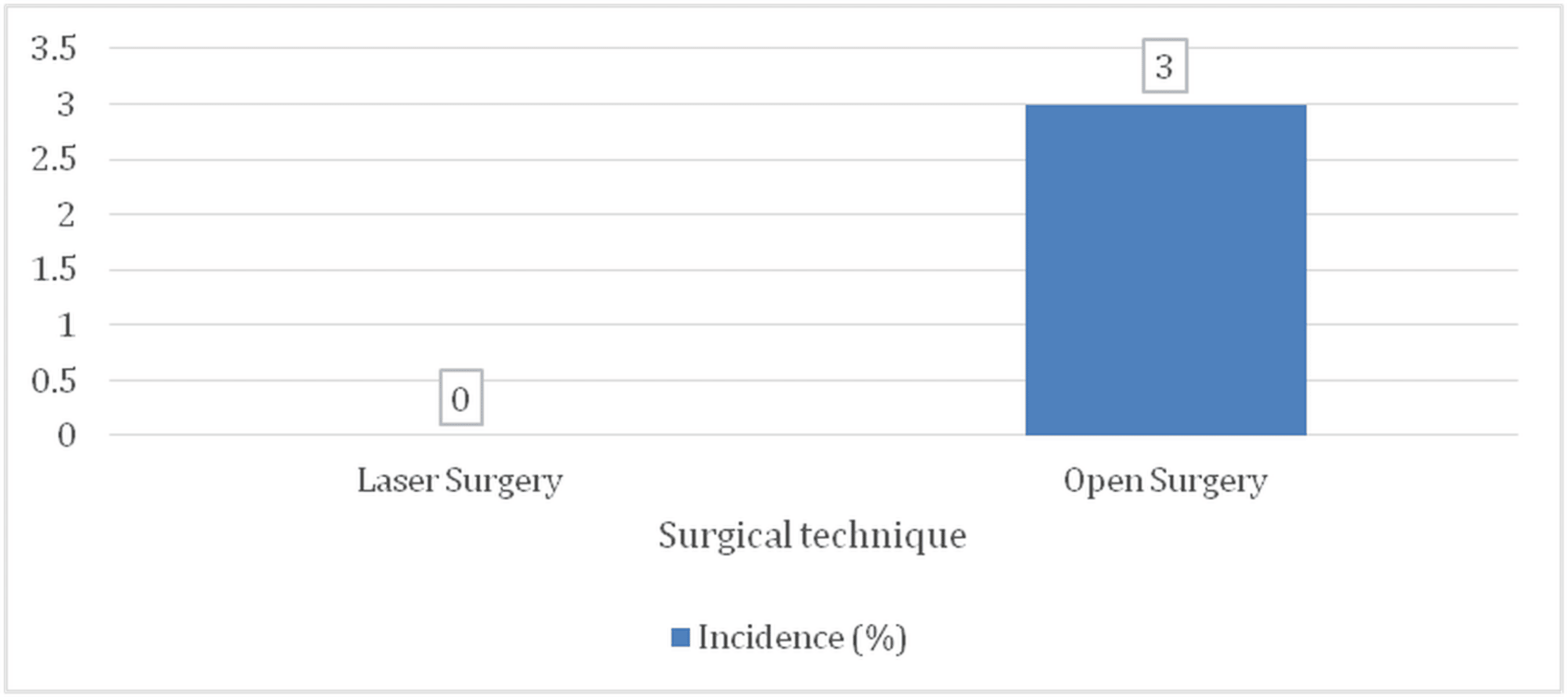
Bar chart for the incidence of anal stenosis (N = 100)

Anal stenosis was observed in one patient (2%) in the open surgery group, and none (0%) of the laser-treated patients (Figure 9). Although this difference was not statistically significant (p = 0.245), it underscores the tissue-preserving advantage of laser treatment.

Wound infection

The incidence of wound infection was significantly lower in the laser group, affecting two patients (5%), compared to seven patients (14%) in the open surgery group. This difference reached statistical significance (p = 0.034) and supports the minimally invasive nature of laser treatment in reducing postoperative morbidity.

Reoperation requirement

Reoperation was required in 15 patients (30%) in the laser surgery group compared to nine patients (18%) in the open surgery group. Although the difference was not statistically significant (p = 0.16), the higher rate in the laser group points to a potential need for secondary intervention in cases with incomplete tract ablation.

Patient satisfaction scores

The mean patient satisfaction score was slightly higher in the open surgery group (6.98 ± 1.96) compared to the laser group (6.34 ± 2.29). Logistic regression analysis identified two significant predictors of poor postoperative outcomes, regardless of the surgical modality: the presence of multiple tracts (odds ratio (OR) = 2.5; p = 0.03) and associated abscess cavities (OR = 3.1, p = 0.02). These findings highlight the importance of fistula complexity and infection burden in influencing treatment success.

To further identify predictors of poor surgical outcomes, a logistic regression analysis was performed (Table 5). The results demonstrated that patients with multiple fistulous tracts had significantly higher odds of recurrence or incomplete healing, with an OR of 2.5 (p = 0.03). Additionally, the presence of associated abscesses was independently associated with poor outcomes, increasing the likelihood of surgical failure by 3.1 times (OR = 3.1; p = 0.02). These findings underscore the importance of preoperative evaluation for tract complexity and abscess formation when planning the surgical approach.

## Discussion

This RCT compared endoscopic laser ablation with conventional open surgical techniques in the management of fistula-in-ano. Laser surgery demonstrated early postoperative benefits such as shorter hospital stays (mean: 1.5 ± 0.5 days) compared to open surgery (mean: 3.8 ± 1.2 days), and lower postoperative pain scores - reported by 35 of 50 patients (70%) in the laser group, versus 15 of 50 patients (30%) in the open group. However, these short-term advantages were counterbalanced by poorer long-term outcomes. Recurrence was observed in 15 patients (30%) undergoing laser surgery compared to nine patients (18%) in the open group, with reoperation rates mirroring these figures (30% vs. 18%).

Earlier studies reported more favorable results for laser-based interventions. Tümer et al. [14] demonstrated better healing times and reduced postoperative morbidity with laser techniques. Lalhruaizela et al. [15] also noted favorable outcomes using endofistula laser ablation in low-complexity tracts. These reports underline the benefit of laser in early recovery phases, especially in simple fistulas.

However, anatomical complexity significantly influences surgical outcomes. The classification proposed by Parks et al. [16] continues to serve as a reliable framework for stratifying fistula types and guiding treatment strategies. In our study, laser treatment failed to provide adequate control in complex presentations, likely due to its limited ability to address secondary tracts and associated abscesses. This is consistent with findings from Sluckin et al. [17], who, in a multicenter retrospective study, found no significant advantage of FiLaC™ over conventional methods, particularly in transsphincteric and suprasphincteric tracts.

At the three-month follow-up, complete healing was documented in 41 of 50 patients (82%) in the open group vs. 35 of 50 patients (70%) in the laser group. Patient satisfaction scores were also higher in the open surgery group (6.98 ± 1.96) compared to the laser group (6.34 ± 2.29), suggesting that long-term healing may outweigh short-term comfort for many patients.

Complication rates were low in both groups, with no reports of anal incontinence or stenosis in the laser cohort. In contrast, the open surgery group had two cases (4%) of mild fecal incontinence and one case (2%) of anal stenosis. While these differences were not statistically significant, they reflect the slightly higher morbidity of open procedures.

Multivariate logistic regression in our study identified key predictors of poor outcomes. Patients presenting with multiple fistulous tracts (eight in the laser group vs. 12 in the open group) had a 2.5-fold increased risk of recurrence, while those with associated abscess formation demonstrated a 3.1-fold increase in the risk of incomplete healing. These findings are supported by earlier works of Parks et al. [16] and Hammond [18], both of whom highlighted the influence of fistula anatomy and infection status on treatment success.

Open surgical approaches such as fistulectomy (performed in 60% of open group patients), fistulotomy (20%), and LIFT (20%) enable complete excision of primary and secondary tracts and facilitate drainage, making them more effective in complex cases. In contrast, laser treatment targets only the internal lumen and may fail to address lateral extensions or undrained abscesses. Emile et al. [19] echoed these limitations in a meta-analysis of minimally invasive techniques such as video-assisted anal fistula treatment (VAAFT), emphasizing that, although such procedures are associated with lower complication rates, they may not ensure definitive healing.

Future directions

Future research should focus on long-term follow-up to assess late recurrences beyond the standard three-month window, as these may influence the true efficacy of each intervention. Cost-effectiveness analysis is also warranted, particularly in light of the higher reoperation rates associated with laser surgery. Exploring hybrid techniques, such as combining laser ablation with procedures such as LIFT or the use of biologic glue, may offer improved outcomes in select cases. Additionally, the integration of patient-reported outcome measures (PROMs) could provide valuable insights into long-term quality of life and continence preservation, enabling more patient-centered care.

Study limitations

This study has several limitations. Being conducted at a single tertiary care center may limit the generalizability of the findings. The three-month follow-up duration might not adequately capture late recurrences or long-term functional outcomes. The relatively small sample size (100 patients) restricts detailed subgroup analysis and may limit the statistical power of certain comparisons. Outcome measures such as postoperative pain and patient satisfaction were self-reported, introducing potential response bias. Additionally, the choice of open surgical technique was based on a surgeon's discretion and fistula anatomy, resulting in intra-group variability. Healing was assessed clinically without the use of imaging modalities such as MRI or endoanal ultrasound, which may affect diagnostic accuracy. Finally, the study did not include a cost analysis, limiting insights into the economic implications of the procedures, particularly laser surgery.

## Conclusions

This RCT found that both laser and open surgical techniques are safe and effective options for managing fistula-in-ano. Open surgery demonstrated better long-term healing and lower recurrence, with higher overall patient satisfaction, although it carried a slightly greater risk of minor complications. Laser surgery offered advantages in terms of faster recovery, less postoperative discomfort, and shorter hospital stay but was associated with a higher need for reoperation, especially in complex fistulas. Surgical choice should be tailored to individual patient factors, fistula anatomy, and surgeon expertise, balancing the benefits of quicker recovery against the potential for recurrence.
